# Supplementary material for: From sequence to enzyme mechanism using multi-label machine learning
Source: BMC Bioinformatics. 2014 May 19;15:150. doi: 10.1186/1471-2105-15-150 (PMC4229970; doi:10.1186/1471-2105-15-150)
Supplement: Additional file 2 — Java code of ml2db. Additional file ml2db_code.tar.gz contains the Java source code to run the multi-label machine learning experiments and save the results to database. The code’s Javadoc is included. [file 1471-2105-15-150-S2.zip › additional file 2/ml2db/ml2db/doc/main/ml2db/package-use.html]

Uses of Package main.ml2db


JavaScript is disabled on your browser.


- Overview
- Package
- Class
- Use
- Tree
- Deprecated
- Index
- Help

- Prev
- Next

- Frames
- No Frames

- All Classes

# Uses of Package main.ml2db

- Packages that use main.ml2db

  | Package | Description |
  | --- | --- |
  |  |  |
  | --- | --- |
  | main.ml2db |  |
  | main.ml2db.al.learning |  |
  | main.ml2db.mulan.attributesfiltered |  |
  | main.ml2db.mulan.learn |  |
  | main.ml2db.mulan.learn.crosseval |  |
  | main.ml2db.mulan.learn.traintest |  |
  | main.ml2db.mulan.mulanarff |  |
  | main.ml2db.mulan.mulanarff.database |  |
  | main.ml2db.weka |  |
  | test.ml2db |  |
  | test.ml2db.alltests |  |
  | test.ml2db.dataharness |  |
  | test.ml2db.weka |  |
- Classes in main.ml2db used by main.ml2db

  Class and Description

  DbManagers

  Stores a database connection to the databases containing: 1.
- Classes in main.ml2db used by main.ml2db.al.learning

  Class and Description

  DbManagers

  Stores a database connection to the databases containing: 1.
- Classes in main.ml2db used by main.ml2db.mulan.attributesfiltered

  Class and Description

  DbManagers

  Stores a database connection to the databases containing: 1.
- Classes in main.ml2db used by main.ml2db.mulan.learn

  Class and Description

  DbManagers

  Stores a database connection to the databases containing: 1.

  Serializer

  Class
- Classes in main.ml2db used by main.ml2db.mulan.learn.crosseval

  Class and Description

  DbManagers

  Stores a database connection to the databases containing: 1.
- Classes in main.ml2db used by main.ml2db.mulan.learn.traintest

  Class and Description

  DbManagers

  Stores a database connection to the databases containing: 1.
- Classes in main.ml2db used by main.ml2db.mulan.mulanarff

  Class and Description

  DbManagers

  Stores a database connection to the databases containing: 1.
- Classes in main.ml2db used by main.ml2db.mulan.mulanarff.database

  Class and Description

  DbManagers

  Stores a database connection to the databases containing: 1.
- Classes in main.ml2db used by main.ml2db.weka

  Class and Description

  DbManagers

  Stores a database connection to the databases containing: 1.
- Classes in main.ml2db used by test.ml2db

  Class and Description

  DbManagers

  Stores a database connection to the databases containing: 1.
- Classes in main.ml2db used by test.ml2db.alltests

  Class and Description

  DbManagers

  Stores a database connection to the databases containing: 1.
- Classes in main.ml2db used by test.ml2db.dataharness

  Class and Description

  DbManagers

  Stores a database connection to the databases containing: 1.

  ProjectParameters

  Contains file paths (database connection files, arff properties files, mulan
  xml files etc.)
- Classes in main.ml2db used by test.ml2db.weka

  Class and Description

  DbManagers

  Stores a database connection to the databases containing: 1.

- Overview
- Package
- Class
- Use
- Tree
- Deprecated
- Index
- Help

- Prev
- Next

- Frames
- No Frames

- All Classes
